# Chronotypes in the US – Influence of age and sex

**DOI:** 10.1371/journal.pone.0178782

**Published:** 2017-06-21

**Authors:** Dorothee Fischer, David A. Lombardi, Helen Marucci-Wellman, Till Roenneberg

**Affiliations:** 1 Department of Environmental Health, Harvard T.H. Chan School of Public Health, Boston, Massachusetts, United States of America; 2 Center for Injury Epidemiology, Liberty Mutual Research Institute for Safety, Hopkinton, Massachusetts, United States of America; 3 Institute for Medical Psychology, Centre of Chronobiology, Ludwig-Maximilian-University, Munich, Germany; Morehouse School of Medicine, UNITED STATES

## Abstract

An individual’s chronotype reflects how the circadian system embeds itself into the 24-h day with rhythms in physiology, cognition and behavior occurring accordingly earlier or later. In view of an increasing number of people working at unusual times and linked health and safety risks, the wide range in human chronotypes may provide opportunities to allow people to work (and sleep) at times that are in synch with their circadian physiology. We aimed at estimating the distribution of chronotypes in the US population by age and sex. Twelve years (2003–2014) of pooled diary data from the American Time Use Survey were used to calculate chronotype based on mid-point of sleep on weekends (MSF^We^, n = 53,689). We observed a near-normal distribution overall and within each age group. The distribution’s mean value is systematically different with age, shifting later during adolescence, showing a peak in ‘lateness’ at ~19 years, and shifting earlier thereafter. Men are typically later chronotypes than women before 40, but earlier types after 40. The greatest differences are observed between 15 and 25 for both sexes, equaling more than 50% of the total chronotype difference across all age groups. The variability in chronotype decreases with age, but is generally higher in males than females. This is the first study to estimate the distribution and prevalence of individual chronotypes in the US population based on a large-scale, nationally representative sample. Our finding that adolescents are on average the latest chronotypes supports delaying school start times to benefit their sleep and circadian alignment. The generally wide range in chronotypes may provide opportunities for tailored work schedules by matching external and internal time, potentially decreasing long- and short-term health and safety risks.

## Introduction

The human circadian system actively synchronizes (entrains) to the 24-h day via environmental signals of light and darkness. Individuals entrain differently depending on exogenous (*i*.*e*., light exposure) and endogenous (*i*.*e*., circadian response characteristics) factors that produce different phenotypes, known as chronotypes [1]. Chronotype refers to the phase of entrainment of an individual, reflecting how the circadian system embeds itself into the 24-h day. Rhythms in physiology, cognition and behavior reach their peaks and valleys accordingly earlier or later, thereby creating individual temporal niches that can be separated by as much as ten hours [2]. These differences are apparent in rhythms of sleep and wake with late chronotypes exhibiting later sleep times, the ability to extend their sleep into the day (*e*.*g*., after a night shift), as well as more flexible day-to-day sleep times [3].

An increasing number of people are working unusual hours. Currently 30% of the U.S. workforce has to alternate, rotate or extend shifts, or follow on-call duty [4]. These unusual work schedules are linked with increased risks for health and safety [5–8]. Finding ways to foster a safe and healthy environment for *all workers* is both a legal and a social responsibility. The wide range in chronotypes in the population may provide opportunities to synch workers to their individual optimal working times that align external demands and internal rhythms, thereby minimizing potentially adverse health effects. Vetter *et al*. [9] demonstrated that sleep and circadian alignment improved in a rotating shift schedule that was tailored to employees’ individual chronotypes. These findings may translate into long-term health benefits: another study by Vetter *et al*. [10] showed in a cohort of 64,615 nurses that the mismatch of chronotype and working time (*i*.*e*., late types working daytime schedules) significantly increased the risk for type 2 diabetes. However, to better inform interventions, we need to understand the population distribution of chronotypes, as well as to what extent chronotype varies by age for males and females. Previous studies on this topic are scarce, and mainly only cover rather small samples (n < 1,000).

The first large-scale study to describe age- and sex-related differences in human chronotypes was conducted by Roenneberg and colleagues in 2004 [11]. Based on an Internet survey of ~25,000 individuals in predominantly German-speaking countries, they described distinct age and gender-related patterns: Children were most frequently early chronotypes with adolescent age groups being progressively later and showing a maximum in ‘lateness’ at around the age of 20. After 20, chronotype was earlier again with increasing age. Roenneberg and colleagues also reported women to be earlier chronotypes than men for most of adulthood; however, this difference disappeared after the age of 50. In a separate study of 8,972 individuals from Northern Italy (age range 10–85 years), Tonetti and colleagues reported similar ages for the peak in ‘lateness’ in men (~21 y) and women (~17 y), as well as the absence of chronotype differences between the sexes beyond age 55 [12]. In 2011, Randler [13] obtained a similar pattern of results for the age range of 12–23 years in a sample of 7,480 participants in Germany: the change from delaying (‘becoming a later chronotype’) to advancing (‘becoming an earlier chronotype’) was apparent in both sexes (around the age of 21), yet this turn occurred earlier in females than males. Furthermore, while the delaying proportion was very rapid and pronounced, the turn back to advancing was smoother. Another population-based study in Finland (n = 10,503, aged 25–74 years) showed similar results: with advancing age, the proportion of early chronotypes increased and the proportion of late types decreased [14].

Only two studies of comparable size were conducted outside Europe. Paine and colleagues showed in a sample of 2,526 New Zealand adults that participants aged 30–34 years were more likely to be definite evening types and less likely to be moderate or definite morning types compared to the age group 45–49 years [15]. Duarte and colleagues assessed diurnal preferences in a sample of 14,650 Brazilians (5-year age bins from <20 to ≥60) [16]. Compared to Roenneberg *et al*. [11] and Tonetti *et al*. [12], they observed a different interaction between age and sex: although women were also earlier chronotypes than men up to the age of 30, this was reversed in age groups older than 45 (no significant sex-differences were found from 30 to 45 years of age). The inconsistencies in chronotype by age and gender between the European vs. Brazilian studies might be population-specific, *e*.*g*., geographical location and the associated variations in sunrise, sunset and day length are important factors for entrainment of the circadian clock, and thus for chronotype [17]. They may also be due to the use of different instruments to assess chronotype. While Duarte *et al*. applied the MEQ (Morningness-Eveningness-Questionnaire [18]), Roenneberg *et al*. and Tonetti *et al*. both used the MCTQ (Munich ChronoType Questionnaire [19]). The MEQ asks about the preferred daily timing for a range of activities (*e*.*g*., physical exercise, eating) producing an arbitrary score, whereas the MCTQ asks and utilizes the actual sleep-wake times on work and work-free days to determine chronotype by the mid-point of sleep on days off.

No study to date has described the distribution and influencing factors (*i*.*e*., age, sex) of chronotypes on a nationally representative scale in the United States or North America. Our aim was therefore to close this gap and provide estimates of the distribution of chronotypes (based on mid-sleep times) in a large, nationally representative US population sample using diary data from the American Time Use Survey (ATUS).

## Materials and methods

### Study data

For our study sample, we pooled data over a period of 12 years (2003–2014) from the American Time Use Survey (ATUS), which is an annual probability-weighted, cross-sectional and nationally representative survey used to measure how, where, and with whom Americans spend their time. Each month, approximately 2,100 people (aged 15 years or older) are randomly selected from a subset of households that have completed their eighth and final month of interviews for the Current Population Survey, which is the primary source of labor force statistics for the United States. Response rates for the ATUS were between 50% and 58% for 2003–2014. Respondents participate in an interview via telephone, after first completing a structured component that gathers information on demographics, work, and home life characteristics.

In a separate diary component, respondents are asked to provide start and end times of every activity they participated in during the 24-hour period prior to the interview. Respondents are asked to recall and report every activity they participated in from 4:00 am the day before the survey through 4:00 am on the day of the survey. Since most people are sleeping at 4:00 am, the end times for the final activity are also recorded (allowing the total duration of the final activity, which is sleep in many cases, to be determined). We included respondents that reported being employed (“at work” or “absent”), unemployed (“on layoff” or “looking”) or retired in the last week, and excluded those not in the labor force due to disability because of a higher prevalence of sleep disturbances in people with disabilities [20].

### Chronotype calculation

An individual’s chronotype can be assessed by mid-sleep on work-free days (MSF) [1]. Mid-sleep is calculated as the halfway point between sleep onset and sleep offset, for example, assuming an 8h sleep duration, a chronotype of 4:00 falls asleep at midnight and wakes up at 8:00 the next day. Work-free days (instead of workdays) are used to reduce the influence of external demands on sleep-wake behavior. Mid-sleeps on workdays tend to be earlier than those on days off, because many people end their sleep prematurely using alarm clocks to get up in time for work [2]. Mid-sleep on work-free days is therefore seen as a more accurate proxy for when sleep is supposed to occur naturally, and has been shown to correlate well with dim-light melatonin onset, a sleep-facilitating hormone under regulation of the circadian clock [21–23].

Optimally, chronotype is calculated from sleep times where sleep offset (waking up) occurs on a work-free day with no use of alarm clocks. While ATUS allows for determining whether or not the day of the diary was a work-free day, no such information is available for the following day. It is thus unknown if sleep ended on a workday or a day off, or if people woke up by themselves or if they were woken up. To circumvent this limitation, we assumed that the likelihood for work-free days is highest on weekends (Saturday/Sunday), and therefore only used mid-sleep times from respondents that completed the diary component on a Friday or a Saturday (meaning that sleep offset was on either a Saturday or a Sunday). Accordingly, for the purposes of this study, chronotype was assessed by the mid-point of sleep on weekends (MSF^We^). Although unemployed and retired respondents may have no workdays per definition, the social structure is often different on weekdays vs. weekends. Chronotype calculation was therefore based on only Fri/Sat diary days for all labor force subgroups (*i*.*e*., employed, unemployed, retired) to maximize their comparability.

Since respondents may report several sleep bouts per 24 hours including naps during the day, we considered only the bout with the longest duration within each individual and excluded sleep bouts that occurred during the daytime (*i*.*e*., mid-sleeps between 12:00 and 20:00). Of these, only sleep bouts with durations between 3h and 14h were considered for chronotype calculation in order to minimize the risk of including respondents suffering from insomnia or hypersomnia or due to data error.

### Data analyses

Data handling, processing and analyses were conducted in SAS/STAT^®^ and R [24]. Mid-sleeps were plotted by age and sex, and a polynomial smoothing function (6^th^ order) was fitted in R. To test for the influence of age and sex on chronotype, a multiple linear regression model was computed with age, sex and their interaction term age*sex as independent variables, adjusted for labor force status (employed (at work, absent) vs. unemployed (on layoff, looking, retired)), diary day (Friday vs. Saturday) and diary month (Jan–Dec), and duration of the sleep bout that was used for mid-sleep calculation. T-tests, respectively their non-parametric equivalent, were performed for 2-group comparisons (*e*.*g*., females *vs*. males in the age group 15-19y). We report mean values and standard deviations within age bins and by sex but given the cross-sectional design of the study (no repeated measures), standard deviations are not available when reporting difference values, *e*.*g*., magnitude of change in chronotype from age 15 to 20. For analysis purposes, mid-sleep times were linearized, transforming mid-sleeps between 20:00 and 24:00 into values between -4.00 and 0.00. All analyses are weighted using the appropriate ATUS weights, which compensate for the survey's oversampling of certain demographic groups, the oversampling of weekend day diaries, and differential response rates across demographic groups. IRB approval was not needed because this was a public database study and no participants were involved. The ATUS multi-year data files used in this study can be downloaded from the ATUS database: https://www.bls.gov/tus/datafiles_0314.htm. The webpage also provides a detailed User’s Guide with information about variable names, data coding, editing, weights, etc. Information to contact the ATUS staff with help requests can be found here: https://www.bls.gov/tus/-contact.

## Results

### Sample description

**Fig 1:**
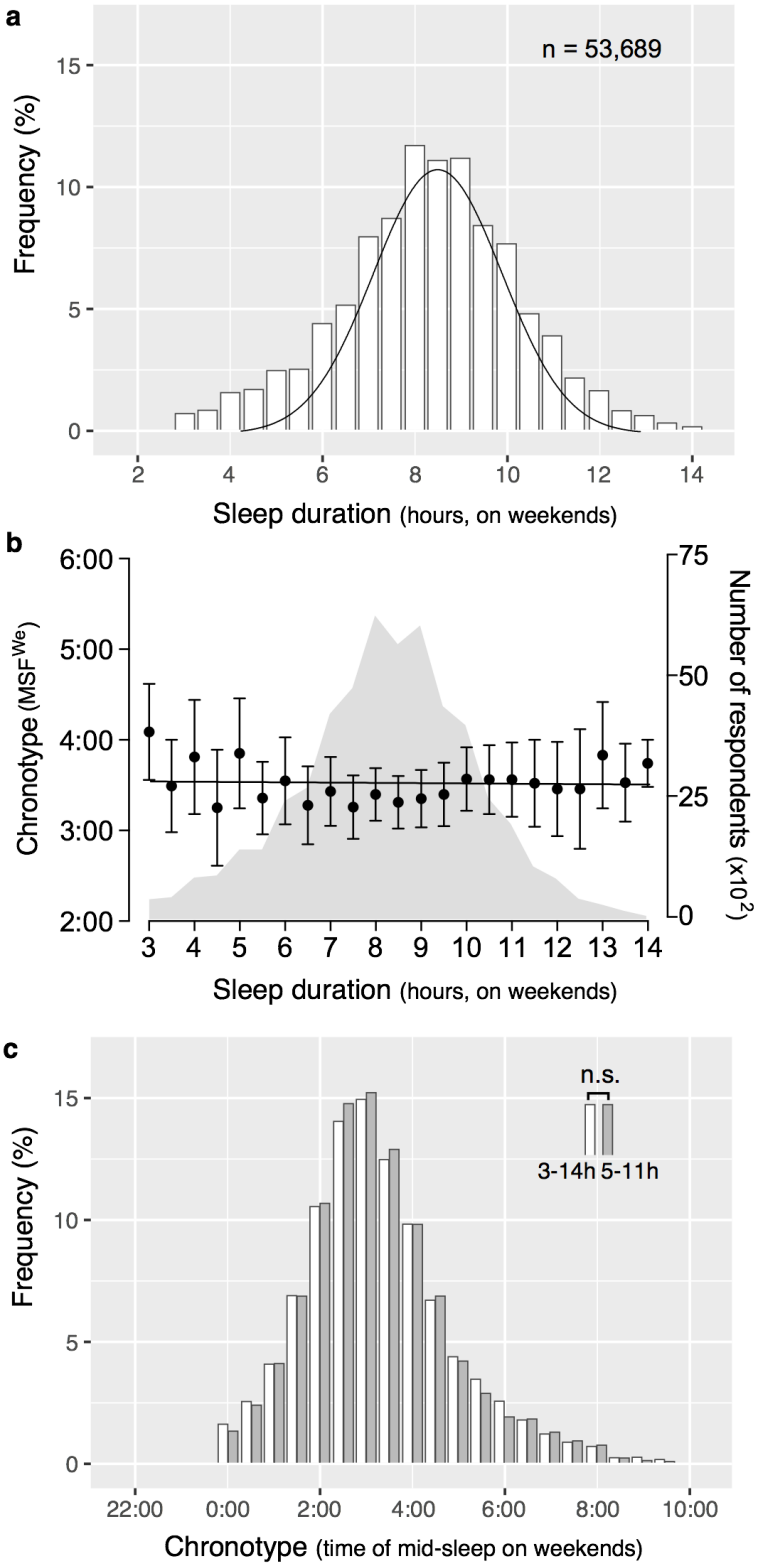
Chronotype and sleep duration. **a)** Distribution of sleep duration on weekends (min. 3h, max. 14h) in the total sample of 53,689 respondents. **b)** Relationship between chronotype (mid-sleep on weekends, MSF^We^) and sleep duration on weekends (mean values and standard errors). No significant association was observed in a univariate regression model (b = -0.0004, p > 0.05), indicating that short and long sleepers are equally frequent among early and late types. **c)** Chronotype distributions did not statistically differ when restricting sleep duration on weekends to 5–11 hours (n = 47,435) *vs*. 3–14 hours (n = 53,689). n.s. = non-significant, *i*.*e*. p > 0.05.

The ATUS is a federally administered survey interviewing respondents in detail about their activities (including sleep times) during the last 24 hours. Of initially 55,075 respondents in the ATUS (pooled over a 12-year period) that reported sleep on a Friday to Saturday or Saturday to Sunday (weekend sleep), 53,689 (97.5%) were considered evaluable based upon our inclusion criteria to analyze their chronotype (assessed by mid-point of sleep on weekends, MSF^We^, see Methods for inclusion criteria). Among the 53,689 persons in the MSF^We^ group, there were 30,226 (56.3%) females and 23,463 (43.7%) males, of which 63.2% were employed (“at work” or “absent”), 5.0% were unemployed (“on layoff” or “looking”), and 31.8% were retired. Average sleep duration was 8.1h ±1.5h, showing a bell-shaped distribution with few respondents sleeping very short (4.6% less than 5h) or very long (5.8% more than 11h) (Fig 1a).

### Distribution and variability of chronotype

**Fig 2:**
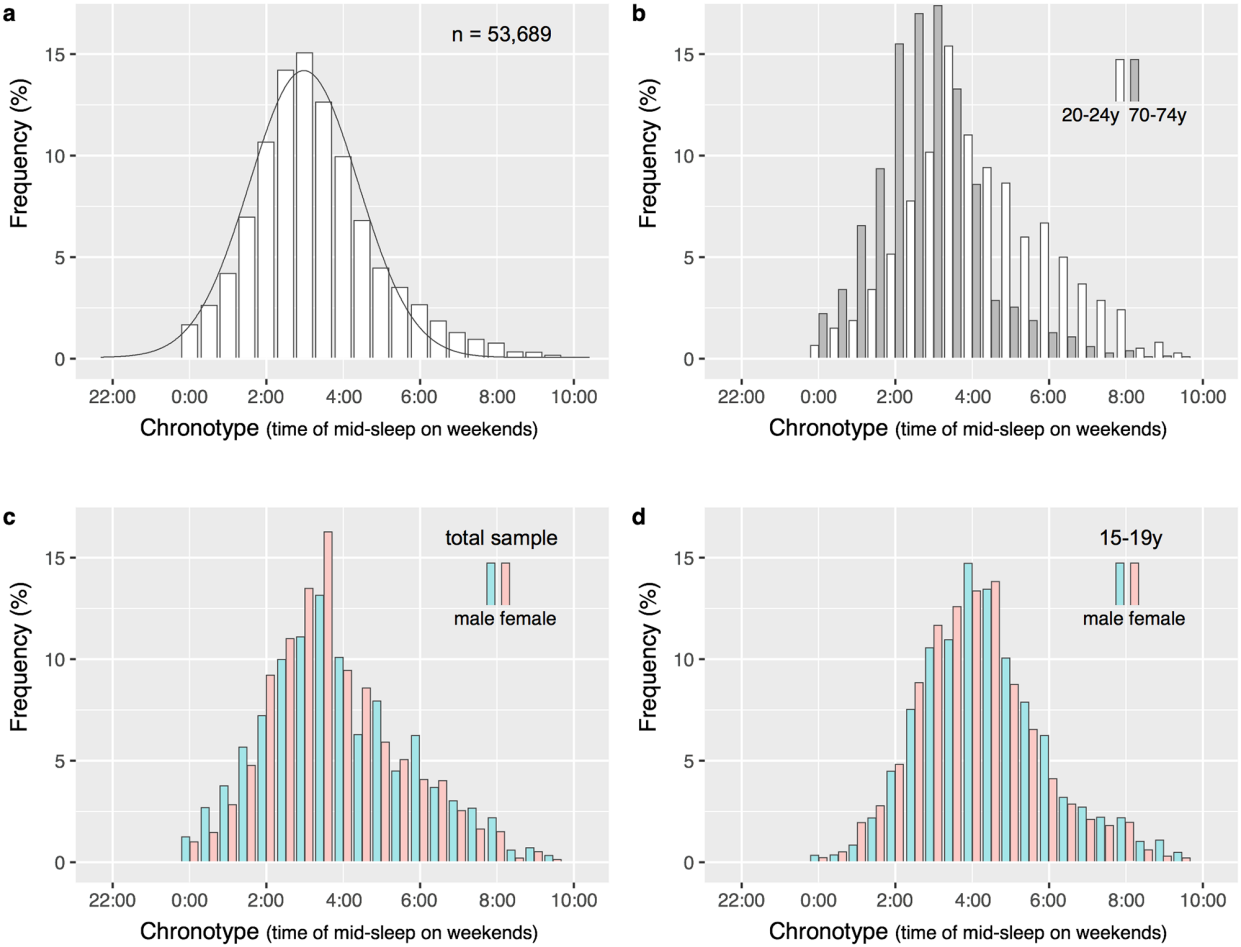
Chronotype distributions (based on weighted frequencies of mid-sleep times on weekends, MSF^We^) in a) the total sample of 53,689 respondents, b) for the age groups 20–24 years (n = 2,479) and 70–74 years (n = 2,266), c) separately for females (n = 30,226) and males (n = 23,463), and d) for males (n = 1,809) and females (n = 1,698) in the age group 15–19 years. Although bell-shaped at all ages, older age groups show leptokurtic distributions with the distribution average shifted to the left (earlier chronotypes) (panel b). Extreme early (MSF^We^ < 2:00) and extreme late (MSF^We^ > 6:00) chronotypes are more frequent in men than women (panel c). While frequencies are higher for girls than boys (15-19y) among early chronotypes (*i*.*e*., MSF^We^ < 3:00), they are higher for boys among late types (*i*.*e*., MSF^We^ > 6:00), overall resulting in a slightly advanced chronotype for girls in this age group (MSF^We^_females_ = 4:24 ±1.97h *vs*. MSF^We^_males_ = 4:31 ±1.99h, p = 0.051) (panel d).

**Fig 3:**
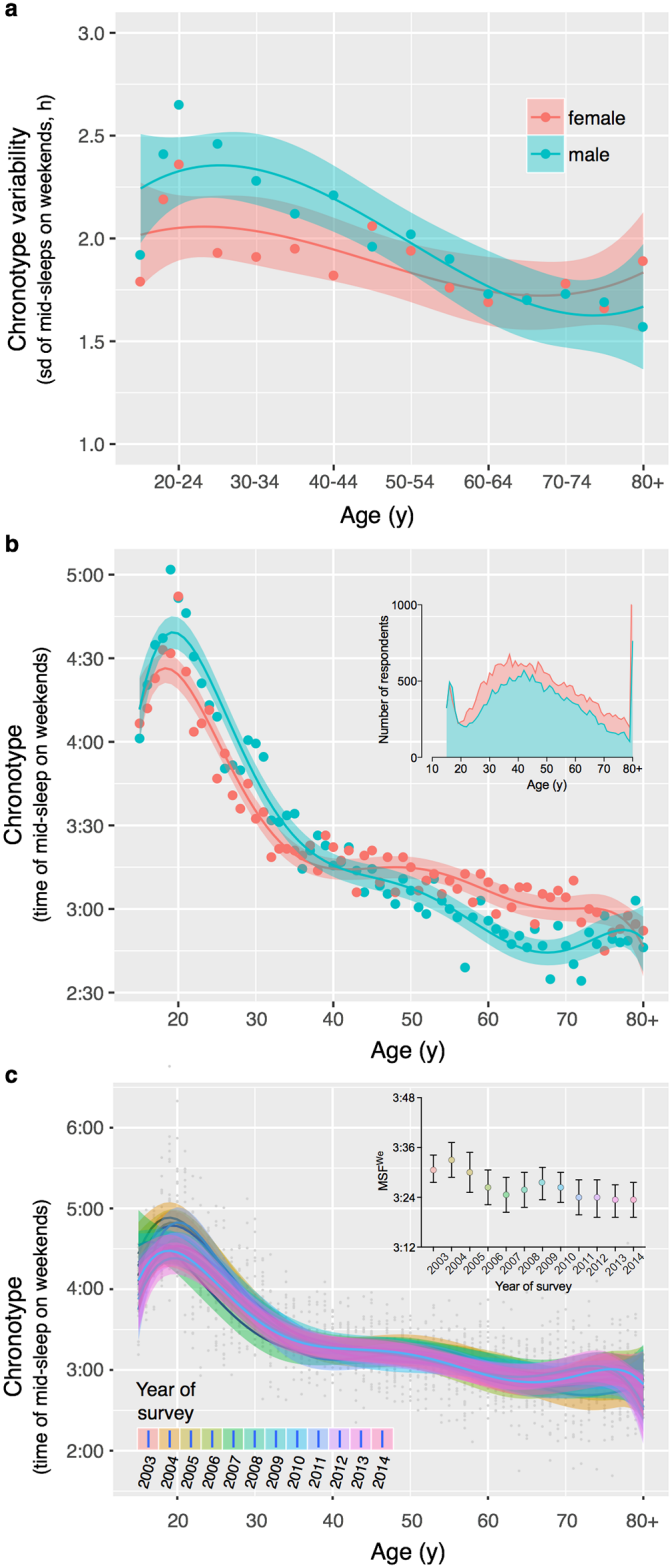
Influence of age and sex on chronotype. Although very early (based on mid-sleep on weekends, MSF^We^ < 2:00) and very late (MSF^We^ > 6:00) chronotypes are observed at all ages and for both sexes, the variability (assessed as sd = standard deviation, panel **a**) as well as the average of chronotype (panel **b**) changes non-linearly with age and sex. The inlay graph in panel b shows the number of respondents at each age by sex; note that one data point is outside the axis limits: women aged 80+ (n = 1602). **c)** The distinct age- and sex-pattern is observed in every year of survey (2003–2014), with a non-significant trend towards slightly earlier distribution means in later years (see inlet in panel c).

The overall distribution of chronotypes (MSF^We^, see Methods for calculation details) follows a near-normal distribution with a slight positive skew (Fig 2a). The range of chronotypes in our sample spans almost 10 hours (MSF^We^_earliest_ = 0:00, MSF^We^_latest_ = 9:53). Twenty-five percent show a chronotype earlier than 2:24, 50% fall between 2:24 and 4:15, and another 25% show a chronotype later than 4:15. Chronotype showed no relationship with sleep duration (univariate regression model: b = -0.0004, p > 0.05). Variance in chronotype appeared slightly higher for short and long sleep durations but the number of respondents was also much lower for these durations (Fig 1b). Importantly, we observe this bell-shape not only for the overall sample but also within each age group (5-year bins), meaning that extreme early and late chronotypes exist at every age (see Fig 2b for comparison of age groups 20-24y and 70-74y). However, the variability in chronotype (measured as standard deviation of mid-sleep times) is different according to age: increased during adolescence and largest in the age group 20–24 years (2.36h in women and 2.65h in men), differences in chronotype are decreased for older age groups showing a minimum variation of 1.66h in women between 75–79 years and 1.57h in men at 80 and older (Fig 3a). Accordingly, men show a greater variability in mid-sleep times than women before the age of 60–64 indicating that extreme chronotypes at both ends are more frequent in men than women for most of their lives (see Figs 2c and 3a). Performing a sensitivity analysis restricting sleep durations to 5-11h (instead of 3-14h) did not change the findings: MSF^We^_3-14h_ = 3:26 ±2.31h *vs*. MSF^We^_3-14h_ = 3:25 ±2.26), p > 0.05 (see Fig 1c).

### Influence of age and sex on chronotype

Chronotype shows a distinct relationship with age and sex (Fig 3b): progressively later MSF^We^ are observed in both men and women during childhood and adolescence reaching a peak in ‘lateness’ at the age of 18.4 years (MSF^We^ = 4:27) in females and 19.2 years in males (MSF^We^ = 4:40) (based on smoothing functions; maxima of average values are much later: 4:52 and 5:02, respectively) (see also Fig 2d for comparison of males and females in the age group 15–19 years). This peak marks a sharp turn from increasingly later to increasingly earlier MSF^We^ with advancing age, showing similar chronotypes in seniors and children (MSF^We^_70y_ = 2:49, MSF^We^_10y_ = 3:09; extrapolated data). The linear slope is steepest for both sexes between 15 and 20, equaling an average difference (delay) in chronotype of 0.76h in girls and 1.01h in boys (b_females_ = 0.14h/8min per age group, b_males_ = 0.18h/11min per age group). The subsequent advance to a similar chronotype of age 15 takes approximately the same time in women but about twice as long in men (b_females_ = -0.26h/16min per age group, b_males_ = -0.12h/7min per age group). Importantly, chronotype differences are largest between the ages 15 and 25, where we see a total difference of 1.85h in women (0.76h delay and 1.09h advance) and of 1.89h in men (1.01h delay and 0.88h advance). Across all age groups in our sample, the chronotype range (MSF^We^_latest_−MSF^We^_earliest_) spans 2.12h in women and 2.46h in men but equals a total difference (adding delay and advance proportions) of 2.88h and 3.47h, respectively. In other words, 64% (females), respectively, 55% (males) of this total difference is observed in the age groups 15–25 years. The difference between males and females is largest at 21.6 years (0.27h/16min, based on smoothing functions) and disappears at 41.4 years (2^nd^ intercept), only to become evident again shortly thereafter. While after the peak gradually earlier MSF^We^ are observed in both sexes with increasing age (groups 20-70y), women show a rather long plateau, with little or no difference in chronotype between 35 and 55. The plateau is also observed in men but for a much shorter period (40-50y). After the age of 41, women show on average a later chronotype than men (0.26h/16min), reversing the pattern prevailing before the age of 41. This sex difference disappears again at 79.1 years (3^rd^ intercept). Average mid-sleep times are shown in Table 1 for all ages separately for men and women.

**Table 1:** Chronotypes^1^ in the US by age and sex based on diary data from the American Time Use Survey (ATUS).

	Women			Men			Total sample		
Age	N	MSF^We^	SD	N	MSF^We^	SD	N	MSF^We^	SD
15	327	4.11	1.71	325	4.02	1.53	652	4.06	1.62
16	476	4.20	1.81	494	4.34	1.79	970	4.27	1.80
17	373	4.38	1.84	458	4.58	2.23	831	4.49	2.07
18	299	4.55	2.21	303	4.62	2.14	602	4.58	2.19
19	223	4.53	2.15	229	5.03	2.60	452	4.78	2.42
20	234	4.87	2.41	214	4.86	2.39	448	4.87	2.40
21	241	4.42	1.90	204	4.77	2.25	445	4.59	2.10
22	297	4.06	2.49	201	4.51	2.43	498	4.27	2.50
23	321	4.11	2.12	224	4.35	3.26	545	4.23	2.92
24	316	4.19	2.52	227	4.22	2.64	543	4.20	2.61
25	365	3.78	2.30	247	4.15	2.52	612	3.97	2.49
26	428	3.93	1.88	273	3.84	2.24	701	3.89	2.10
27	483	3.68	1.83	306	3.86	2.36	789	3.77	2.23
28	499	3.60	1.71	344	3.83	2.16	843	3.71	2.01
29	479	3.75	1.83	345	4.01	2.84	824	3.88	2.58
30	554	3.54	1.98	387	3.99	2.53	941	3.78	2.46
31	537	3.58	2.04	443	3.91	2.40	980	3.75	2.30
32	601	3.31	1.92	417	3.53	1.69	1,018	3.41	1.85
33	611	3.36	1.86	431	3.52	2.07	1,042	3.44	1.99
34	612	3.36	1.65	479	3.56	2.16	1,091	3.46	1.96
35	599	3.35	1.68	474	3.57	1.93	1,073	3.45	1.83
36	621	3.32	1.91	479	3.24	1.79	1,100	3.28	1.86
37	675	3.38	2.04	524	3.35	2.31	1,199	3.36	2.20
38	594	3.23	1.80	502	3.44	2.15	1,096	3.33	1.98
39	633	3.44	2.20	502	3.38	2.27	1,135	3.41	2.24
40	599	3.37	1.88	531	3.26	2.20	1,130	3.31	2.07
41	615	3.29	2.00	528	3.28	2.14	1,143	3.29	2.09
42	600	3.35	1.59	571	3.37	2.04	1,171	3.36	1.84
43	596	3.10	1.84	539	3.23	2.36	1,135	3.17	2.15
44	617	3.32	1.74	501	3.10	2.18	1,118	3.21	2.01
45	600	3.35	2.38	541	3.24	2.03	1,141	3.30	2.21
46	554	3.16	2.01	495	3.14	1.98	1,049	3.15	2.00
47	626	3.31	2.25	492	3.09	1.99	1,118	3.21	2.15
48	597	3.10	1.60	487	3.03	2.05	1,084	3.06	1.86
49	557	3.31	1.89	431	3.18	1.67	988	3.26	1.79
50	550	3.25	1.84	447	3.11	2.00	997	3.18	1.93
51	540	3.11	1.86	471	3.01	1.98	1,011	3.06	1.94
52	500	3.17	1.88	453	2.97	1.85	953	3.07	1.87
53	483	3.21	2.13	418	3.18	2.08	901	3.20	2.11
54	470	3.09	1.89	418	3.05	2.13	888	3.07	2.04
55	477	3.17	1.61	391	3.00	1.98	868	3.09	1.81
56	492	3.12	1.81	373	2.95	1.73	865	3.04	1.78
57	463	3.21	1.78	393	2.65	1.76	856	2.93	1.86
58	473	3.04	1.76	374	2.95	1.95	847	2.99	1.87
59	466	3.21	1.81	359	3.05	1.95	825	3.13	1.90
60	412	3.16	1.67	350	2.93	1.75	762	3.04	1.73
61	403	2.97	1.81	345	2.88	1.77	748	2.92	1.80
62	413	3.12	1.62	313	2.85	1.70	726	2.99	1.69
63	406	3.01	1.69	295	2.79	1.63	701	2.90	1.68
64	364	3.13	1.64	279	2.84	1.78	643	2.99	1.73
65	394	3.13	1.71	308	2.77	2.17	702	2.96	1.98
66	366	2.91	1.54	282	2.88	1.39	648	2.90	1.47
67	360	3.09	1.67	266	2.78	1.54	626	2.95	1.64
68	350	3.07	1.99	216	2.58	1.52	566	2.85	1.80
69	298	3.11	1.61	222	2.90	1.69	520	3.01	1.66
70	271	3.07	1.59	216	2.78	1.50	487	2.92	1.56
71	286	3.17	2.03	172	2.67	1.68	458	2.96	1.95
72	289	2.92	1.57	165	2.57	1.74	454	2.77	1.70
73	267	3.00	1.97	162	2.86	2.05	429	2.94	2.04
74	279	2.98	1.61	159	2.79	1.50	438	2.91	1.58
75	258	2.75	1.55	149	2.96	2.00	407	2.83	1.78
76	249	2.86	1.82	160	2.82	1.38	409	2.84	1.64
77	262	2.88	1.72	165	2.80	1.20	427	2.85	1.50
78	228	2.96	1.72	129	2.81	2.08	357	2.90	1.87
79	196	2.91	1.29	104	3.05	1.89	300	2.97	1.60
80+	1,602	2.87	1.89	761	2.77	1.57	2,363	2.83	1.77
Total	30,226	3.42	2.14	23,463	3.45	2.44	53,689	3.43	2.31

MSF^We^, mid-sleep on weekends; SD, standard deviation.

^1^Chronotype is assessed by MSF^We^ based on one-day diary data. Times are shown as decimal values, *i*.*e*., 4.50 equals 4:30 (average values).

Computing a multiple regression model adjusted for labor force status, sleep duration, and day and month of the diary confirms the significant impact of age (b = -0.21h, p < 0.001), sex (b = -0.31h, p < 0.001) and their interaction term (b_age*sex_ = 0.05h, p < 0.001) on chronotype (Table 2). Sleep duration, diary day and labor force status are also statistically significant factors (b = -0.04, b = 0.22h and b = 0.08h, all p < 0.001): mid-sleep times are on average 2 minutes earlier for every hour more sleep, 13 minutes later for sleep between Saturday and Sunday than for sleep between Friday and Saturday, and 5 minutes later for unemployed compared with employed respondents.

**Table 2:** Adjusted regression model for the impact of age and sex on chronotype^1^.

Variable	b	se	t	p
Intercept	3.603	0.101	35.77	<.0001
Age (y)	-0.215	0.006	-36.33	<.0001
Sex (referent: male)	-0.308	0.027	-11.38	<.0001
Age*Sex	0.046	0.004	12.54	<.0001
Sleep duration (h)	-0.044	0.003	-12.79	<.0001
Labor force status (referent: employed)	0.075	0.004	19.94	<.0001
Day of diary (referent: Friday)	0.219	0.013	16.59	<.0001
Month of diary	0.001	0.002	0.49	0.627

b, unstandardized regression coefficient; se, standard error.

^1^Chronotype is assessed by mid-sleep times on weekends (MSF^We^) based on one-day diary data.

To test whether the year of the survey might have influenced our results (*i*.*e*., cohort effect), we examined the relationship of chronotype with age and sex by survey year (2003–2014). The same pattern was observed in every year, showing a peak in adolescent groups and earlier MSF^We^ in older age groups (Fig 3c). Later survey years (2011–2014) showed a trend towards earlier chronotypes on average based on smaller peaks (earlier MSF^We^) in adolescent groups but this was not significant (linear regression model; survey year: b = -0.001, year*age: b = -0.0002, year*sex: b = -0.016, year*age*sex = 0.002, all p > 0.05).

## Discussion

This is the first study to estimate the distribution of individual chronotypes in the US population based on diary data in a large-scale, nationally representative sample from the American Time Use Survey. Self-reported sleep times were used to calculate mid-sleeps on weekends as a proxy for chronotype (MSF^We^). A near-normal distribution is observed both overall and in each age group indicating that very early and very late chronotypes are present at all ages, however mean values change systematically across the lifespan. Chronotype becomes later in both sexes throughout adolescence, reaching a peak in ‘lateness’ at ~18 years for women and ~19 years for men. Men delay faster between the ages of 15 and 20 and advance more slowly between 20 and 40, resulting in a later chronotype on average than women during this period. This pattern is reversed beyond 40 years of age, when men show earlier chronotypes than women (by approximately the same magnitude of ~15min). More than 50% of the lifelong chronotype change occurs during adolescence and early adulthood, while variability decreases with age.

### Adolescents are on average the latest chronotypes

Our findings are generally consistent with previous studies showing a peak in lateness during late adolescence/ early adulthood and a steady advance in chronotype thereafter [11–13, 16]. This marked turn has been proposed as a marker for the “end of adolescence” suggesting the involvement of hormones interacting with the circadian system [11]. Young people, including senior high school students, are on average the latest chronotypes in society, and their delayed sleep-wake rhythms clash with school start times resulting in pronounced discrepancies between sleep-wake behavior and associated physiological rhythms. They produce a strong weekly structure in sleep-wake behavior alternating between short and early sleep during the school week and long and late recovery sleep on weekends. This is essentially the same phenomenon as jetlag that occurs when traveling across time zones, and has even been termed “social jetlag” [25, 26]. According to the CDC, 82.3% of public schools in the US (with an estimated total enrollment of 4.2 million students) start school at 8:30 a.m. or earlier [27]. Our study shows an average chronotype of 4:30 a.m. in the group of 17- and 18-year olds, suggesting that senior high school students get up and go to school in their biological night. Sleep plays a vital role in adolescents’ physical and mental health, and performance [28–30], and the American Academy for Sleep Medicine recommends 8–10 hours of sleep on a regular basis for adolescents aged 13 to 18 to promote optimal health [31]. Our findings suggest that adolescents’ chronotypes are on average too late for school start times before 8:30 a.m., *i*.*e*., many may not be able to advance their sleep enough to get sufficient sleep, which is in line with another CDC report showing that 68.8% of US high school students sleep less than 8h during an average school night [32]. Evidence is mounting that one simple and effective (yet not the only) way to benefit adolescent sleep is to delay school start times [33].

### Men and women are different chronotypes–depending on age

Previous studies have reported the disappearance of a sex difference in chronotype around the age of 50 [11, 12] and attributed this to changes in the endocrine system, coinciding with the average onset of menopause in women. In contrast to their findings, and in line with Duarte et al. [16], we observed an interaction between sex and age: women were on average earlier chronotypes than men until the age of 40 but later types thereafter. Importantly, women over 40 were later chronotypes relative to men, but not relative to women before the age of 40. This reversed chronotype difference between males and females over 40 in our data is mainly due to the rather long plateau in the chronotype profile of females (between 35–50 years). Hormonal changes might act as modulators for an aging circadian system, causing a slow-down in women between 35 and 50 (and a speed-up in men aged 55 to 65). Studies examining circadian differences between males and females usually report an earlier phase of core body temperature (CBT) and melatonin rhythms in women than men [34–38]. These studies, conducted with younger subjects (<30yrs), are consistent with our results and previous findings that suggest that women are earlier chronotypes than men during adolescence and early adulthood. Only one study looked at circadian period (estimated from CBT and melatonin data) in both younger (<35yrs) and older (>50yrs) individuals observing no interaction between age and sex: women had, on average, a shorter period than men in both age groups [39]. Although circadian period is one factor impacting upon phase (shorter periods result in earlier phases [40]), other factors such as circadian amplitude might account for further variance [41]. Differences in chronotype between females and males as well as among different age groups may also have non-hormonal causes, *e*.*g*., differences in family life (*e*.*g*., typically-‘female’ responsibilities such as household chores and childcare), work regimes (*e*.*g*., shift work) as well as somatic and mental disorders (*e*.*g*., depression [14]), that may feed back onto the circadian clock via modification of internal bodily processes and/or light-dark exposure (‘zeitnehmer loop’ [42]).

### Chronotype differs largely among individuals at any age but is less variable in older individuals and women

The large differences in chronotype are up to 10h among individuals of any age, consistent with previous studies (*e*.*g*., [2]). The average chronotype for people between 25 and 65 years lies between 3:58 (±2.49h) and 2:58 (±1.98h), and assuming 8-h sleep need, optimal sleep for these chronotypes falls between 0:00 and 8:00, respectively, between 23:00 and 7:00. Assuming 35min for morning routine and taking into account an average US commute time of 25min [43], a worker requiring 8h of sleep would need to be as early as 3:00 a.m. for a work start time of 8:00 a.m. Our findings show that as much as 60% have a later chronotype, and probably suffer from at least some amount of social jetlag and sleep loss. The extent may vary with age, sex, social responsibilities, job schedule (*i*.*e*., night and shift work) and importantly, individual sleep need (*i*.*e*., similar to chronotype, sleep duration shows a large inter-individual variance [2, 44], as was also observed in this study) but is likely to be the highest among adolescents.

Although the wide range of chronotypes can be observed across all ages, the variability in chronotype decreases with increasing age (resulting in leptokurtic distributions in older age groups). This might be due to age-related differences in the likelihood of sleep at different circadian phases meaning that the range of times at which older individuals can readily sleep is narrower, thus limiting the variance in chronotype [45]. However, again this observation could be related to less variability in social responsibilities such as when individuals get older there may be less variability in what they need to get up for on a weekend (such as taking care of children and children’s activities). The men in our sample showed on average a greater range of chronotypes (MSF^We^_latest_−MSF^We^_earliest_) as well as a greater range of chronotype variability (sd_largest_−sd_smallest_) than women, suggesting that their sleep-wake behavior (and probably circadian system) might be more variable. Two studies looked at sex differences in circadian amplitude, and found lower melatonin amplitude in males than females [36, 46]. Lower amplitude may reflect a weaker intrinsic oscillator, thus accounting for a higher variability in the output, *i*.*e*., phase of behavioral rhythms. However, this finding is inconsistent across studies as one study reported that the amplitude of CBT was larger in men than women [36].

### Sleep duration, employment status and diary day influence chronotype

With each additional hour of sleep, chronotype was 2 minutes earlier; among unemployed respondents, chronotype was on average 5 minutes later than that of employed respondents. Both effects were significant but are negligible differences (note that sleep duration was significant only when adjusted for other variables, including age and sex). When chronotype (MSF^We^) assessment was based on Fri-Sat sleep, mid-sleep times were significantly later by 13 minutes than for Sat-Sun sleep. Assuming the majority of people work weekdays and have weekends off, this may be due to Fri-Sat sleep being influenced by work on Friday: getting up earlier for work increases the time awake and thus homeostatic sleep pressure, advancing sleep onset and accordingly mid-sleep times. Moreover, because of Friday being a workday for many people, Saturdays are often used for social events offering a night between two days off. A study assessing dim-light melatonin onset (DLMO), considered a phase marker of the circadian master clock, showed that DLMO delayed progressively over the weekend [47]. Although mid-sleep times derived from Saturday diaries seem better suited for determining chronotype, we argue MSF^We^ derived from Friday diaries is still a more accurate chronotype estimate than mid-sleep on weekdays (MSW). Both sleep on- and offset used to calculate MSW are influenced by workdays while it is mainly sleep onset for MSF^We^ on Fridays with the opportunity to sleep in on Saturdays.

### Limitations

One important limitation of our data is the lack of information regarding the next day after the diary day (*i*.*e*., workday/work-free day and use of alarm clocks/wake-up mode). This may have resulted in an over-estimation of early types in our sample. Indeed, when choosing cut-offs from earlier studies to classify chronotypes as early (<3:00), intermediate (3:00–5:00) and late (>5:00) [2], we find 40% early, 44% intermediate and only 16% late chronotypes. Cut-offs are sensitive to the study population (*e*.*g*., race/ethnicity [48, 49]) and other differences need to be examined, such as latitude and longitude [50], position within a time zone [17], and work schedules (day work vs. shift work) [51, 52]. Thus, cut-offs should be interpreted with caution.

The response rate in the ATUS was only fair (50–58%) with no information about non-responders and may indicate there could be time use bias as we might expect that those who are busier may not have as much time available to participate. However, we note that the distribution of hours worked and hours slept in the ATUS is overall representative of the national population, as reported elsewhere [53], and therefore we believe that any non-response bias would have little effect on our analyses. Furthermore, because we excluded respondents not in the labor force due to disabilities, the study sample is not nationally representative of the general population but representative of employed, retired and non-disabled unemployed respondents.

Mid-sleep times calculated from self-reported sleep times may be subject to recall bias or individual rounding to systematic increments. Sleep bouts used for chronotype calculation were restricted to durations between 3h and 14h to minimize the risk of including people suffering from sleep disorders or data errors, however this may lead to misclassification, *e*.*g*., short sleep durations that reflect work-related sleep (before or after a shift). This would result in an over-estimation of extreme early or late chronotypes. Likewise, because information about previous sleep is not available in the ATUS, we could not apply the mid-sleep correction for sleep loss on workdays [26], which may have resulted in over-estimating the proportion of late chronotypes. Yet, given that the distribution of chronotypes in our study is highly similar to previous findings and that our findings did not change when restricting sleep durations to 5-11h, we argue that the extent of misclassification was minimal overall.

We interpret the age-specific chronotype pattern as changes over time as have studies before ours [11, 16] but given the cross-sectional nature of the data we cannot exclude that the actual age course might be different. Middle-term evidence for an age effect comes from a study that followed adolescents for 2.5 years showing that DLMO indeed delayed over this period [54]. First long-term support is provided by Broms and colleagues who analyzed the age-standardized chronotype of 567 male individuals in Finland, assessed in 1985 and 2008 [55]. They found that in most individuals, chronotype shifted towards ‘more morningness’, *i*.*e*., 66% of evening types and 47% of somewhat evening types advanced. They also found that chronotype changed in only very few from clearly evening to clearly morning and vice versa, suggesting that the relative position of an individual within the distribution is fairly stable. Furthermore, because of the cross-sectional study design and the limited survey period of twelve years, we cannot distinguish a cohort effect from an age effect. There is some evident that the proportion of late chronotypes has grown larger over the past decades [26, 55], possibly due to the increased use of light-emitting devices such as laptops and smartphones. Their use especially in the evening, and thus exposure to blue-enriched light after dark, has been shown to be associated with melatonin suppression, and delayed circadian phase and sleep patterns [56, 57]. Nevertheless, Roenneberg et al. [11] found a similar age-specific chronotype profile when looking at yearly entries in their large database between 2003 and 2012, providing some evidence for an age- vs. cohort- effect. We confirmed their finding in our study, observing the same age- and sex-pattern for each year of survey (2003–2012).

### Conclusions

Given the cross-sectional design of all large-scale chronotype studies, longitudinal data collection is needed to characterize individual trajectories. Future studies looking at working time, sleep and health are encouraged to include a chronotype measure to account for potential effect modification, especially in samples with great age spans. Our finding that adolescents on average are the latest chronotypes of all age groups argues for debate on delayed school start times that may benefit sleep and circadian alignment. The generally wide range in chronotypes of up to 10h is both challenge and opportunity in a 24/7-society. Great diversity implies that not everyone can be accommodated but contrary to “one size fits all”-approaches, it also provides opportunities for flexible work arrangements and tailored shift schedules that make use of individuals’ temporal niches to increase the match between work and circadian time, and thus decrease long and short-term health and safety risks.
